# Evaluation of Amphetamine-Related Hospitalizations and Associated Clinical Outcomes and Costs in the United States

**DOI:** 10.1001/jamanetworkopen.2018.3758

**Published:** 2018-10-19

**Authors:** Tyler N. A. Winkelman, Lindsay K. Admon, Latasha Jennings, Nathan D. Shippee, Caroline R. Richardson, Gavin Bart

**Affiliations:** 1 Division of General Internal Medicine, Department of Medicine, Hennepin Healthcare, Minneapolis, Minnesota; 2 Hennepin Healthcare Research Institute, Minneapolis, Minnesota; 3 Department of Obstetrics and Gynecology, University of Michigan Medical School, Ann Arbor; 4 Institute for Healthcare Policy and Innovation, University of Michigan, Ann Arbor; 5 Division of Health Policy and Management, School of Public Health, University of Minnesota, Minneapolis; 6 Department of Family Medicine, University of Michigan Medical School, Ann Arbor; 7 Division of Addiction Medicine, Department of Medicine, Hennepin Healthcare, Minneapolis, Minnesota

## Abstract

**Importance:**

Despite indications of increasing amphetamine availability and psychostimulant deaths in the United States, evidence across data sources is mixed, and data on amphetamine-related hospitalizations are lacking.

**Objective:**

To clarify trends in amphetamine-related hospitalizations and their clinical outcomes and costs in the United States.

**Design, Setting, and Participants:**

This repeated, cross-sectional study used hospital discharge data from the Healthcare Cost and Utilization Project National Inpatient Sample. The nationally representative sample included US adults (n = 1 292 300) who had amphetamine-related hospitalizations between January 1, 2003, and December 31, 2015. Multivariable logistic and Poisson regression models were used to examine in-hospital mortality and length of stay. Analysis of these data was conducted from November 2017 to August 2018.

**Exposure:**

Amphetamine dependence or abuse or amphetamine poisoning.

**Main Outcomes and Measures:**

Annual hospitalizations, in-hospital mortality, length of stay, transfer to another facility, and costs.

**Results:**

Over the 2003 to 2015 study period, there were 1 292 300 weighted amphetamine-related hospitalizations. Of this population, 541 199 (41.9%) were female and 749 392 (58.1%) were male, with a mean age of 37.5 years (95% CI, 37.4-37.7 years). Amphetamine-related hospitalizations, compared with other hospitalizations, were associated with age younger than 65 years (98.0% vs 58.0%; *P* < .001), male sex (60.3% [95% CI, 59.7%-60.8%] vs 41.1% [95% CI, 40.9%-41.3%]), Medicaid coverage (51.2% [95% CI, 49.8%-52.7%] vs 17.8% [95% CI, 17.5%-18.1%]), and residence in the western United States (58.5% [95% CI, 55.9%-61.0%] vs 18.9% [95% CI, 18.0%-19.8%]). Amphetamine-related hospitalizations declined between 2005 and 2008, and then increased from 55 447 hospitalizations (95% CI, 44 936-65 959) in 2008 to 206 180 hospitalizations (95% CI, 95% CI, 189 188-223 172) in 2015. Amphetamine-related hospitalizations increased to a greater degree than hospitalizations associated with other substances. Adjusted mean length of stay (5.9 [95% CI, 5.8-6.0] vs 4.7 [95% CI, 4.7-4.8] days; *P* < .001), transfer to another facility (26.0% [95% CI, 25.3%-26.8%] vs 18.5% [95% CI, 18.3%-18.6%]; *P* < .001), and mean in-hospital mortality (28.3 [95% CI, 26.2-30.4] vs 21.9 [95% CI, 21.6-22.1] deaths per 1000 hospitalizations; *P* < .001) were higher for amphetamine-related than other hospitalizations. Annual hospital costs related to amphetamines increased from $436 million (95% CI, $312 million-$559 million) in 2003 to $2.17 billion (95% CI, $1.95 billion-$2.39 billion) by 2015.

**Conclusions and Relevance:**

Given that amphetamine-related hospitalizations and costs substantially increased between 2003 and 2015, pharmacologic and nonpharmacologic therapies for amphetamine use disorders and a coordinated public health response are needed to curb these rising rates.

## Key Points

**Question:**

What are the current trends in frequency and costs of amphetamine-related hospitalizations in the United States?

**Findings:**

In this cross-sectional study of approximately 1.3 million amphetamine-related US hospitalizations between 2003 and 2015, hospitalizations increased substantially by 2015, with the highest frequency being in the western United States and the predominant payer being Medicaid.

**Meaning:**

Amphetamine use may be an emerging public health issue; pharmacologic and nonpharmacologic therapies that effectively treat amphetamine use disorder are needed.

## Teaser

This cross-sectional study examines Healthcare Cost and Utilization Project National Inpatient Sample data from 2003 to 2015 to identify the frequency, outcomes, and cost of hospitalizations associated with amphetamine use in the United States.

## Introduction

Largely overshadowed by the opioid epidemic, deaths from psychostimulants increased more than 250% between 2008 and 2015 after a period of declining amphetamine use.^1,2^ Use of amphetamines, primarily illicit methamphetamine hydrochloride, surged in the early 2000s and then began to decline in the mid-2000s as precursors to methamphetamine production were restricted.^3^ Coinciding with an uptrend in psychostimulant deaths are increases in the demand and supply of high-purity, low-cost methamphetamine, as the US Drug Enforcement Administration recently reported.^1^ In some US states, deaths from methamphetamine have surpassed deaths from heroin.^4^ Preliminary evidence shows that amphetamine-related use of treatment facilities and emergency departments (EDs) has increased in the past several years.^5,6,7^ Amphetamine use is now the fourth most common reason to seek drug treatment in the United States, after alcohol, opioid, and marijuana use.^5^

Nationally representative surveys, designed to be the key source for substance use prevalence in the United States, have not reported a concurrent increase in methamphetamine use among community-dwelling individuals.^8^ For example, national survey data indicate that methamphetamine use declined between 2002 and 2014 for individuals aged 18 years or older.^9,10^ The discrepancy between survey results and other data sources has created uncertainty for policymakers and health care practitioners who must prioritize a response to amphetamine use relative to other substance use trends. Trends in hospitalizations related to amphetamine use (ie, hospitalizations in which a clinician identified current amphetamine dependence or abuse, or amphetamine poisoning, as one of the issues relevant to the hospital stay) and associated costs have not been defined, but they could serve to clarify important population-level trends in serious amphetamine use and its implications for health.^11^

To shed light on this critical public health topic, we examined 3 key issues using a nationally representative sample of hospitalizations: (1) frequency of amphetamine-related hospitalizations over time; (2) length of stay (LOS), transfer rates to other facilities, and in-hospital mortality among hospitalizations with and without an amphetamine-related diagnosis; and (3) per-episode costs and annual, inflation-adjusted costs associated with amphetamine-related hospitalizations. Hospital inpatient claims are an ideal data source because they include amphetamine use that has been identified by a clinician as problematic, represent cases that are serious enough for hospital admission, and are likely to include populations that are undersampled or excluded from household surveys (eg, homeless or incarcerated individuals).

## Methods

This study was exempt from human participant review, according to the Hennepin Healthcare Research Institute policy on the use of deidentified data sets. We conducted analysis of these data from November 2017 to August 2018. This study followed the Strengthening the Reporting of Observational Studies in Epidemiology (STROBE) reporting guideline.

### Study Design and Setting

We used 2003 to 2015 data from the National Inpatient Sample (NIS), part of the Healthcare Cost and Utilization Project (HCUP) sponsored by the Agency for Healthcare Research and Quality, to conduct a repeated cross-sectional analysis.^12^ The NIS is the largest nationally representative, all-payer database of hospital discharges in the United States, representing 97% of the US population. Short-term rehabilitation hospitals, long-term acute care hospitals (beginning in 2012), psychiatric hospitals, and substance use treatment facilities are not included in the NIS sampling frame.^13^ However, the NIS captures nearly all substance use–related hospitalizations from community hospitals as well as hospitalizations that may subsequently result in stays in short-term rehabilitation or long-term acute care hospitals. To account for sampling changes in 2012, we applied trend weights provided by HCUP to allow for comparison across years.^12,13^

### Sample

The sample included hospitalizations between January 1, 2003, and December 31, 2015, of US adults (aged 18 years or older). We identified amphetamine-related hospitalizations using the *International Classification of Diseases, Ninth Revision, Clinical Modification* (*ICD-9-CM*) codes for amphetamine dependence or abuse (304.40-304.42, 305.70-305.72) or amphetamine poisoning (969.72).^14,15,16^ We excluded diagnostic codes for amphetamine dependence or abuse in remission (304.43 and 305.73) to limit the sample to individuals who were currently using amphetamines. Hospitalizations were defined as amphetamine-related if a diagnosis code for amphetamine dependence or abuse or amphetamine poisoning was listed in any diagnosis position. This approach is consistent with the methods from the Centers for Medicare & Medicaid Services for identifying chronic or potentially disabling conditions in hospital billing data.^17^ Diagnostic codes do not discriminate between methamphetamine use, other illicit amphetamine use, and nonmedical use of prescription amphetamines, but evidence indicates that such codes primarily represent methamphetamine use. Data from ED visits suggest that methamphetamine use composes most of the acute care visits related to amphetamines. For example, methamphetamine accounts for 102 961 (64.4%) of 159 840 amphetamine-related ED visits,^18^ and ED visits associated with methamphetamine are approximately 3 times as prevalent as ED visits for prescription amphetamines (102 961 vs 31 244 visits).^7,19^ In addition, diagnoses (eg, endocarditis) that are common among individuals who inject methamphetamine, but not as prevalent among those with nonmedical use of prescription amphetamines, are associated with amphetamine-related diagnostic codes.^14,20^

We described a number of characteristics of the sample, including age, sex, race/ethnicity, income quartile of patient zip code, hospital census region, other types of co-occurring substance use (eg, alcohol; cannabis; opioids; cocaine; hallucinogens; sedatives, tranquilizers, or hypnotics), and drug-induced mental disorders. We modified the *ICD-9-CM* coding schemes developed by HCUP to define additional substance-related categories (eTable 1 in the Supplement).^21^

We also assessed the 15 most common primary diagnoses among amphetamine-related hospitalizations to illustrate the comorbidities typically associated with amphetamine use. We used *ICD-9-CM* codes to identify hospitalizations with a primary diagnosis of amphetamine dependence or abuse, amphetamine poisoning, or substance use–induced mental disorder. We categorized all other primary diagnoses according to HCUP Clinical Classifications Software for *ICD-9-CM*.^22^

### Outcome Measures

#### Amphetamine-Related Hospitalizations

We estimated the number of amphetamine-related hospitalizations in each study year. In addition, because use of opioids and other substances have also increased during this period,^11,23,24,25,26^ we examined the number of amphetamine-related hospitalizations that were associated with a co-occurring opioid-related diagnosis^21^ or any other substance (eg, alcohol; cannabis; opioids; cocaine; hallucinogens; sedatives, tranquilizers, or hypnotics). Furthermore, to assess the robustness of our main measure specification, we examined hospitalizations with a diagnosis code for amphetamine dependence or abuse or poisoning in the first position only. To explore the geographic burden of amphetamine-related hospitalizations, we assessed the number of such hospitalizations in each year by hospital census region.

We also examined whether trends in amphetamine-related hospitalizations substantively differed from trends in other substance use–related hospitalizations during the study period. We defined each substance using diagnosis codes both in any position and in the first position only to assess the robustness of our results in various specifications.

#### Health Care Utilization, Health Outcomes, and Health Care Costs

Among hospitalizations with and without an amphetamine-related diagnosis in 2014 to 2015, we compared 2 measures of health care utilization: LOS and transfer to another health facility (eg, hospital, psychiatric hospital, addiction treatment center). Because of data limitations, the type of transfer facility could not be disaggregated. We estimated the proportion of hospitalizations that resulted in death and the total number of deaths among amphetamine-related hospitalizations in 2015, the most recent year of data in this study. Per capita hospital costs were also examined.

Finally, we calculated the total annual hospital costs for amphetamine-related hospitalizations by primary payer (ie, all payers, Medicaid, uninsured, Medicare, or private insurance) over the study period.

To obtain hospital costs, we used the Agency for Healthcare Research and Quality Cost-to-Charge Ratio Files to convert charges.^12^ We applied the cost to charge ratio to each charge in the analytic sample and inflation-adjusted resultant costs to 2015 US dollars using the Consumer Price Index for All Urban Consumers.^27^

### Statistical Analysis

We used weighted frequencies to describe the characteristics of hospitalizations with and without an amphetamine-related diagnosis. Differences were compared statistically using Pearson χ^2^ tests.

Weighted counts were used to describe trends in amphetamine-related hospitalizations over time, and we performed subgroup analyses by region. Significance testing was performed with logistic regression, modeling year as a categorical variable.

To compare amphetamine-related hospitalization trends with hospitalization trends for other substance use disorders, we estimated weighted, population-adjusted frequencies (hospitalizations per 100 000 US adults) using US Census Bureau data as the denominator, as recommended by HCUP.^28^ We estimated annual substance-specific hospitalization rates between January 1, 2008, and December 31, 2015. We calculated the percentage change in each substance-specific hospitalization trend between January 1, 2008, to December 31, 2008, and January 1, 2015, to December 31, 2015.

We used multivariable Poisson regression to compare LOS and multivariable logistic regression to compare transfer rates between hospitalizations with and without an amphetamine-related diagnosis. These models accounted for age, sex, race/ethnicity, income quartile of patient zip code, and hospital region.

Similar multivariable logistic regression models were used to compare in-hospital mortality, and multivariable Poisson regression models with robust SEs were used to compare costs per hospitalization between hospitalizations with and without an amphetamine-related diagnosis.

Finally, total annual hospital costs for amphetamine-related hospitalizations were obtained by using the Stata total commands, and subgroup analyses were performed by primary payer.

All analyses were conducted with Stata/MP, version 15.1 (StataCorp LLC). Estimates were weighted, unless otherwise noted, to allow for nationally representative inferences and to account for the 2012 changes to the NIS sampling strategy. In 2015, *ICD-9-CM* diagnosis codes were available only for the first 3 quarters. We adjusted the survey weights in 2015 to generate annualized estimates from the first 3 quarters of data. We considered a 2-sided *P* < .05 to be statistically significant. We followed the 7 research practices recommended when using National Inpatient Sample data^29^ and the STROBE reporting guideline for cross-sectional studies, including clear variable specification, description of statistical analysis, and reporting 95% CIs.^30^

## Results

### Sample Characteristics

The study sample contained 82 491 358 unweighted hospitalizations, representative of 402 942 144 hospitalizations between January 1, 2003, and December 31, 2015. Over the study period, there were 1 292 300 weighted amphetamine-related hospitalizations (unweighted n = 255 507). Of this population, 541 199 (41.9%) were female and 749 392 (58.1%) were male, with a mean age of 37.5 years (95% CI, 37.4-37.7). These hospitalizations, compared with other hospitalizations, were more likely to be associated with age younger than 65 years (98.0% vs 58.0%; *P* < .001), male sex (60.3% [95% CI, 59.7%-60.8%] vs 41.1% [95% CI, 40.9%-41.3%]), and residence in the western United States (58.5% [95% CI, 55.9%-61.0%] vs 18.9% [95% CI, 18.0%-19.8%]) (Table 1). Native American race/ethnicity was 3 times more common among amphetamine-related, compared with other, hospitalizations (2.0% [95% CI, 1.7%-2.3%] vs 0.6% [95% CI, 0.5%-0.7%]); Hispanic race/ethnicity was also more common (16.0% [95% CI,14.9%-17.2%] vs 10.7% [95% CI, 10.2%-11.2%]), whereas African American race/ethnicity was less common (8.7% [95% CI, 8.1%-9.3%] vs 15.1% [95% CI, 14.6%-15.5%]). Medicaid was the predominant payer for amphetamine-related hospitalizations (51.2% [95% CI, 49.8%-52.7%]) but covered a minority of other hospitalizations (17.8% [95% CI, 17.5%-18.1%]).

**Table 1.** Sociodemographic Characteristics and Substance Use Patterns of Study Population, 2014 to 2015

Variable	Weighted, % (95% CI)		*P* Value
	Amphetamine-Related Hospitalizations (n = 64 789)	Other Hospitalizations (n = 10 416 921)	
Male	60.3 (59.7-60.8)	41.1 (40.9-41.3)	<.001
Age, y			
18-25	15.2 (14.7-15.6)	7.6 (7.5-7.7)	<.001
26-40	41.6 (40.9-42.3)	17.7 (17.5-17.9)	
41-64	41.2 (40.4-42.0)	32.7 (32.6-32.9)	
≥65	2.0 (1.9-2.2)	42.0 (41.7-42.3)	
Race/ethnicity			
Non-Hispanic white	68.3 (66.7-69.8)	68.2 (67.4-68.9)	<.001
African American	8.7 (8.1-9.3)	15.1 (14.6-15.5)	
Hispanic	16.0 (14.9-17.2)	10.7 (10.2-11.2)	
Asian/Pacific Islander	2.8 (2.1-3.8)	2.6 (2.5-2.8)	
Native American	2.0 (1.7-2.3)	0.6 (0.5-0.7)	
Other	2.2 (2.0-2.5)	2.9 (2.7-3.1)	
Primary payer			
Uninsured	16.2 (15.2-17.2)	4.4 (4.3-4.6)	<.001
Medicare	14.2 (13.8-14.7)	46.8 (46.5-47.1)	
Medicaid	51.2 (49.8-52.7)	17.8 (17.5-18.1)	
Private	13.8 (13.1-14.5)	28.1 (27.8-28.5)	
Other	4.6 (4.1-5.1)	2.9 (2.8-3.0)	
Median household income quartile			
0-25, poorest	37.2 (35.6-38.7)	30.3 (29.7-31.0)	<.001
26-50	28.2 (27.4-29.1)	26.4 (25.9-26.9)	
51-75	22.0 (21.1-23.0)	23.4 (22.9-23.8)	
76-100	12.6 (11.7-13.6)	19.9 (19.2-20.6)	
US region			
Northeast	3.1 (2.8-3.4)	19.1 (18.1-20.1)	<.001
Midwest	14.6 (13.1-16.2)	22.6 (21.6-23.7)	
South	23.9 (22.0-25.8)	39.4 (38.1-40.6)	
West	58.5 (55.9-61.0)	18.9 (18.0-19.8)	
Alcohol use	25.3 (24.7-25.8)	6.1 (6.0-6.2)	<.001
Cannabis use	28.9 (28.3-29.6)	1.9 (1.9-2.0)	<.001
Opioid use	20.7 (19.9-21.5)	2.1 (2.0-2.1)	<.001
Cocaine use	11.5 (11.0-12.0)	1.1 (1.1-1.2)	<.001
Hallucinogen use	1.7 (1.6-1.8)	0.04 (0.038-0.042)	<.001
Sedative use	7.3 (6.9-7.7)	0.6 (0.57-0.61)	<.001
Other drug use	8.5 (8.1-8.9)	1.4 (1.4-1.5)	<.001
Drug-induced mental disorder	14.3 (13.5-15.0)	0.9 (0.8-0.9)	<.001

One-quarter of amphetamine-related hospitalizations were associated with alcohol (25.3% [95% CI, 24.7%-25.8%]) or cannabis use (28.9% [95% CI, 28.3%-29.6%]) and 1 in 5 (20.7% [95% CI, 19.9%-21.5%]) was associated with opioid use (Table 1).

Substance use and mental health disorders accounted for 9 of the top 15 primary diagnoses among amphetamine-related hospitalizations (eTable 2 in the Supplement). Skin and subcutaneous tissue infections and cardiovascular comorbidities were also common among amphetamine-related hospitalizations.

### Amphetamine-Related Hospitalizations

**Figure 1.**
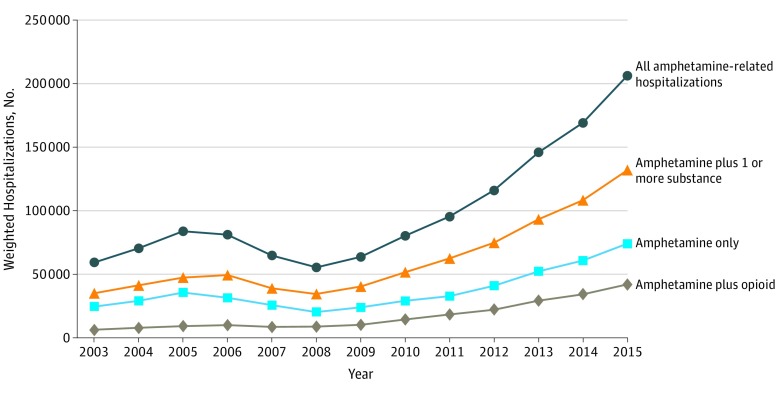
Amphetamine-Related Hospitalizations in the United States, 2003 to 2015

Between 2003 and 2005, the number of amphetamine-related hospitalizations increased from 59 684 (95% CI, 44 784-74 585) to 83 873 (95% CI, 68 936-98 810), consistent with known methamphetamine trends in the mid-2000s.^3^ These hospitalizations declined to 55 447 (95% CI, 44 936-65 959) by 2008 and then increased more than 270% to 206 180 (95% CI, 189 188-223 172) between 2008 and 2015 (Figure 1). More than half of these hospitalizations were associated with at least 1 additional substance throughout the study period. In 2015, 132 107 (64.1%) of 206 180 amphetamine-related hospitalizations were associated with at least 1 additional substance. Similarly, between 2003 and 2015, hospitalizations complicated by both opioids and amphetamines increased 537% from 6705 (95% CI, 4669-8741) to 42 680 (95% CI, 39 181-46 179).

Primary hospital diagnoses related to amphetamine use increased from 1234 (95% CI, 876-1592) in 2008 to 11 780 (95% CI, 10 808-12 752) in 2015, a 9½-fold increase.

**Figure 2.**
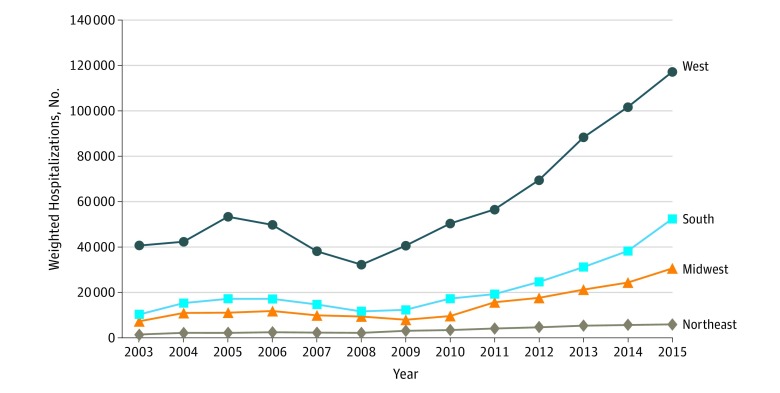
Amphetamine-Related Hospitalizations by US Census Region, 2003 to 2015

Amphetamine-related hospitalizations were substantially higher in the western United States, but more than doubled in all US census regions between 2008 and 2015 (Figure 2). These hospitalizations increased between 2008 and 2015 from 32 388 (95% CI, 23 088-41 688) to 117 213 (95% CI, 102 233-132 194) in the West, from 11 685 (95% CI, 9063-14 308) to 52 460 (95% CI, 46 344-58 576) in the South, from 9325 (95% CI, 5410-13 239) to 30 687 (95% CI, 25 585-35 788) in the Midwest, and from 2049 (95% CI, 1320-2778) to 5820 (95% CI, 4977-6663) in the Northeast.

Compared with changes in other substance–related hospitalizations between 2008 and 2015, amphetamine-related hospitalizations increased to a substantially larger degree (Table 2). For example, hospitalizations with an amphetamine-related diagnosis in any position increased 245.2% (24.1 to 83.2 hospitalizations per 100 000 adults), compared with 45.9% (188.6 to 275.1 hospitalizations per 100 000 adults) for opioid-related hospitalizations and 24.8% (1067.4 to 1331.8 hospitalizations per 100 000 adults) for hospitalizations related to any substance. Trends were similar, although somewhat more exaggerated, when limited to hospitalizations where a substance was identified in the first diagnosis position only.

**Table 2.** Trends in Substance-Related Hospitalizations per 100 000 US Adults, 2008 to 2015

Year	Amphetamine	Alcohol	Cannabis	Cocaine	Hallucinogen	Opioid	Sedative	Other Drug	Any Substance
**Substance Identified in Any Diagnosis Position, Weighted Frequencies**^**a**^									
2008	24.1	690.5	141.6	170.7	3.8	188.6	75.0	117.9	1067.4
2009	27.5	716.5	161.3	166.3	3.7	210.1	76.3	131.9	1126.9
2010	34.2	752.9	177.5	169.7	4.3	231.7	83.6	141.5	1197.5
2011	40.0	746.6	189.4	166.1	5.0	239.1	86.5	159.8	1212.6
2012	48.2	748.5	201.8	149.3	5.1	243.7	83.2	166.3	1223.0
2013	60.1	739.2	212.5	144.4	5.1	248.3	78.5	172.3	1226.0
2014	69.0	746.8	237.7	140.8	5.4	260.9	77.1	179.7	1265.9
2015	83.2	775.4	270.1	146.2	7.3	275.1	77.4	182.9	1331.8
% Change 2008-2015	245.2	12.3	90.7	−14.4	91.0	45.9	3.1	55.1	24.8
**Substance Identified in First Diagnosis Position Only, Weighted Frequencies**^a^									
2008	0.5	137.6	0.7	6.4	0.4	35.7	25.5	4.4	210.7
2009	1.0	138.8	0.8	4.6	0.4	33.7	25.2	4.8	208.1
2010	2.5	147.4	0.9	5.9	0.5	37.2	26.3	5.4	223.6
2011	3.1	140.0	0.7	11.7	0.7	37.5	26.3	4.9	221.8
2012	3.4	137.0	0.8	8.0	0.8	37.4	25.7	5.2	214.9
2013	3.9	133.8	0.7	7.5	0.8	35.5	23.0	5.1	206.3
2014	4.0	138.3	0.8	6.6	1.0	33.2	20.9	5.4	206.2
2015	4.8	145.2	0.8	6.5	2.0	33.2	19.7	5.3	212.7
% Change 2008-2015	786.1	5.6	8.9	1.6	399.6	−7.0	−23.0	21.6	0.9

^a^ Denominator is from the US Census Bureau data provided by the Healthcare Cost and Utilization Project.^28^

### Health Care Utilization, Health Outcomes, and Health Care Costs

Adjusted mean LOS was more than 1 day longer (5.9 [95% CI, 5.8-6.0] vs 4.7 [95% CI, 4.7-4.8] days; *P* < .001) and transfer rates to another facility were statistically significantly higher (26.0% [95% CI, 25.3%-26.8%] vs 18.5% [95% CI, 18.3%-18.6%]; *P* < .001) for hospitalizations related to amphetamines compared with other hospitalizations (Table 3). Adjusted in-hospital mortality was 6.4 (95% CI, 4.4-8.5) deaths per 1000 hospitalizations higher among amphetamine-related, compared with other, hospitalizations (28.3 [95% CI, 26.2-30.4] vs 21.9 [95% CI, 21.6-22.1] deaths per 1000 hospitalizations). In 2015, 2667 deaths (95% CI, 2314-3019) occurred among amphetamine-related hospitalizations.

**Table 3.** Health Care Utilization, In-Hospital Mortality, and Hospital Costs by Amphetamine-Related Hospitalization Status, 2014 to 2015

Outcome^a^	Mean (95% CI)		*P* Value
	Amphetamine-Related Hospitalizations	Other Hospitalizations	
Length of stay, d	5.9 (5.8-6.0)	4.7 (4.7-4.8)	<.001
Transfer to another facility, %	26.0 (25.3-26.8)	18.5 (18.3-18.6)	<.001
In-hospital mortality per 1000 hospitalizations, No.	28.3 (26.2-30.4)	21.9 (21.6-22.1)	<.001
Cost per hospitalization, US$	10 941 (10 615-11 268)	11 737 (11 590-11 884)	<.001

^a^ Adjusted for age, sex, race/ethnicity, zip code income quartile, and hospital region.

Costs per hospitalization were statistically significantly lower among amphetamine-related hospitalizations, compared with other hospitalizations (Table 3), while total hospital costs related to amphetamines increased substantially between 2003 and 2015. Amphetamine-related hospital costs totaled $436 million (95% CI, $312 million-$559 million) in 2003 and increased to $2.17 billion (95% CI, $1.95 billion-$2.39 billion) by 2015. Between 2013 and 2015, the costs of hospitalizations for an amphetamine-related diagnosis covered by Medicaid spiked from $563 million (95% CI, $474 million-$652 million) to $1.25 billion (95% CI, $1.09 billion-$1.40 billion), representing approximately 57.6% of hospital costs for amphetamine-related hospitalizations in 2015. Amphetamine-related hospital costs among the uninsured population decreased from $335 million (95% CI, $294 million-$376 million) in 2013 to $244 million (95% CI, $214 million-$274 million) in 2015.

## Discussion

Amphetamine-related hospitalizations increased between 2008 and 2015 from 55 447 to 206 180 and totaled $2.17 billion in hospital costs by 2015. These hospitalizations more than doubled in every region of the United States, but were most prevalent in the West, and increased to a greater degree than hospitalizations for other substance use disorders. Our findings suggest that amphetamine use may represent an emerging public health issue. Public health responses that address the socioeconomic factors in addiction are needed, particularly because no pharmacologic options exist for the treatment of amphetamine use disorders.^31^

Compared with all other hospitalizations, amphetamine-related hospitalizations were associated with a 29% higher rate of adjusted in-hospital mortality. Higher in-hospital mortality may be associated with the known cardiovascular effects of amphetamine use.^32,33^ From the 2015 NIS data, we identified 2667 deaths during amphetamine-related hospitalizations, while the Centers for Disease Control and Prevention reported 5716 deaths from psychostimulant overdose (excluding cocaine).^26^ Our study did not account for overdose deaths that occurred outside of the hospital and included deaths from possible amphetamine use complications but not overdose. Therefore, the inpatient deaths in this study partially reflect the deaths as defined and reported by the Centers for Disease Control and Prevention, and the study provides additional information about mortality related to psychostimulant use in the United States.

Use of other substances (eg, opioids, alcohol) increased during the study period,^34,35^ but we found substantially larger proportional increases in amphetamine-related hospitalizations compared with hospitalizations for other substance use disorders between 2008 and 2015. This finding suggests that rising levels of amphetamine-related hospitalizations are unlikely to be associated solely with rising rates of substance use–related hospitalizations.

Total LOS was statistically significantly higher among amphetamine-related hospitalizations than other hospitalizations. Rates of transfers to another facility were also more common among amphetamine-related hospitalizations, suggesting that individuals with amphetamine use may require ongoing care and may be unable to return home. We are unaware of previous data that document higher LOS and facility transfers related to amphetamine use.

Amphetamine-related hospital costs were disproportionately incurred by patients covered by Medicaid, particularly beginning in 2014 when Medicaid was expanded through the Affordable Care Act. Some evidence indicates that the costs of medication for opioid use disorders can lower overall costs incurred by Medicaid through lower utilization of nonopioid–related care.^36^ However, to our knowledge, no such data exist for amphetamine use disorders, in part, because effective medication for amphetamine use disorder does not exist.^31^ Behavioral therapies, contingency management in particular, are the only treatments currently available for amphetamine use disorder, but their efficacy is rarely sustained.^37,38,39^ As such, the public health approach proposed by experts to combat the opioid epidemic may also prove useful if expanded to include other substances, such as amphetamines.^40,41^ For example, as is the case for opioid use, needle and syringe exchange programs,^42^ safe-injection facilities,^43^ and a broader mission to address the socioeconomic factors associated with despair and addiction^44^ may reduce the burden of amphetamine abuse as well as other substance use disorders.

### Limitations

These findings should be interpreted within the context of the study’s limitations. As noted, diagnostic codes do not differentiate methamphetamine use from other illicit amphetamine use or nonmedical prescription amphetamine use. However, our findings strengthen the assertion that amphetamine-related hospitalizations are primarily associated with methamphetamine. For example, only 13.8% of amphetamine-related hospitalizations were covered by private insurance. This low coverage rate is similar to the low rates of private insurance among individuals who use methamphetamine (25%)^45^ and varies substantially from the higher rates of private insurance among individuals who use nonmedical prescription amphetamine (65%).^46^ Furthermore, we found that most amphetamine-related hospitalizations occurred in the western region of the United States, which is most consistent with geographic trends in methamphetamine use.^1,3,47^ Use of nonmedical prescription amphetamine is relatively homogeneous across the country.^46^ Nonetheless, our findings may reflect a concurrent rise in hospitalizations for prescription amphetamine users. The lack of specificity of *ICD-9-CM* diagnostic codes has persisted in updated *ICD-10* codes and raises concerns that the current coding structures lack sufficient granularity for detecting and responding to emerging public health issues related to a variety of psychostimulants.

Another limitation is that this study relied on hospital claims data, whose primary purpose is to record billing. Therefore, our findings could reflect changes in billing practices over time, although the trends we identified are similar to other reports of rising methamphetamine use.^1,47^ The NIS sampling frame was redesigned during our study period, which could introduce a break in trends beginning in 2012. However, we applied trend weights to account for these changes. Furthermore, no substantive changes in the NIS sampling frame occurred between 2012 and 2015, the period with the steepest rise in amphetamine-related hospitalizations. Because we used hospital claims data, we did not capture trends in the general population. It is likely that the burden of amphetamines across the US population is substantially larger, if varied in acuity, than that documented in this study of amphetamine-related hospitalizations.

We are limited in our ability to clarify discrepancies between our findings and national survey data. These inconsistencies may be related to the exclusion of populations disproportionately affected by substance use (eg, those who are homeless or incarcerated) or to the underreporting of methamphetamine use because of stigma^48^ in national surveys. On the other hand, amphetamine use may increasingly lead to hospitalization because of changes in amphetamine use risk behavior (eg, injection drug use), drug purity, or adulterants.

## Conclusions

Amphetamine-related hospitalizations in the United States tripled between 2008 and 2015 following a brief period of decline between 2005 and 2008. Most of these hospitalizations were covered by Medicaid and occurred in the western United States. Rising rates of amphetamine use resulted in $2.17 billion in annual hospital costs by 2015 and higher in-hospital mortality rates compared with other hospitalizations. Pharmacologic and nonpharmacologic therapies that effectively treat amphetamine use disorders, as well as a coordinated public health response, are needed to curb rapidly rising rates of amphetamine use.

## Notes

Supplement. **eTable 1.** International Classification of Disease, 9th Edition, Clinical Modification (ICD-9-CM) Codes for Other Substance-Related Categories **eTable 2.** Top 15 Primary Diagnoses Among Amphetamine-Related Hospitalizations, United States - 2014/2015

## References

[1] US Department of Justice Drug Enforcement Administration. *2017 National Drug Threat Assessment* Washington, DC: US Department of Justice Drug Enforcement Administration; 2017 https://www.dea.gov/sites/default/files/2018-07/DIR-040-17_2017-NDTA.pdf. Accessed May 10, 2018.

[2] US Department of Justice Drug Enforcement Administration.*Drugs of Abuse* *A DEA Resource Guide.* Washington, DC: US Department of Justice Drug Enforcement Administration; 2017 https://www.dea.gov/documents/2017/06/15/drugs-abuse. Accessed May 10, 2018.

[3] Maxwell JC, Brecht M-L Methamphetamine: here we go again? Addict Behav. 2011;36(12):-. doi:10.1016/j.addbeh.2011.07.017 21875772 PMC3243901

[4] Ingold J. More Coloradans died last year from drug overdoses than any year in the state’s history: that shows how the opioid epidemic is changing. *Denver Post* April 4, 2018 https://www.denverpost.com/2018/04/04/colorado-drug-overdoses-opioid-deaths-hit-high/. Accessed June 6, 2018.

[5] National Admissions to Substance Abuse Treatment Services. Treatment Episode Data Set (TEDS) 2004-2014. Rockville, MD: Substance Abuse and Mental Health Services Administration; 2016 https://wwwdasis.samhsa.gov/dasis2/teds_pubs/2014_teds_rpt_natl.pdf. Accessed May 10, 2018.

[6] Richards JR, Hamidi S, Grant CD, Methamphetamine use and emergency department utilization: 20 years later. J Addict. 2017;2017:4050932. doi:10.1155/2017/4050932 28913001 PMC5585625

[7] Substance Abuse and Mental Health Services Administration. Emergency department visits involving methamphetamine: 2007 to 2011. *The DAWN Report* June 19, 2014 https://www.samhsa.gov/data/sites/default/files/DAWN_SR167_EDVisitsMeth_06-12-14/DAWN-SR167-EDVisitsMeth-2014.htm. Accessed May 10, 2018. 27606402

[8] Substance Abuse and Mental Health Services Administration. Key substance use and mental health indicators in the United States Results from the 2016 National Survey on Drug Use and Health. Rockville, MD: Center for Behavioral Health Statistics and Quality, Substance Abuse and Mental Health Services Administration; 2017 https://store.samhsa.gov/shin/content//SMA17-5044/SMA17-5044.pdf. Accessed May 10, 2018.

[9] Substance Abuse and Mental Health Services Administration. Results from the 2014 National Survey on Drug Use and Health: detailed tables (Table 7.14B). https://www.samhsa.gov/data/sites/default/files/NSDUH-DetTabs2014/NSDUH-DetTabs2014.htm#tab7-14b. Accessed August 22, 2018.

[10] Substance Abuse and Mental Health Services Administration. Results from the 2014 National Survey on Drug Use and Health: detailed tables (Table 7.14A). https://www.samhsa.gov/data/sites/default/files/NSDUH-DetTabs2014/NSDUH-DetTabs2014.htm#tab7-14a. Accessed August 22, 2018.

[11] Villapiano NLG, Winkelman TNA, Kozhimannil KB, Davis MM, Patrick SW Rural and urban differences in neonatal abstinence syndrome and maternal opioid use, 2004 to 2013. JAMA Pediatr. 2017;171(2):194-196. doi:10.1001/jamapediatrics.2016.3750 27942711

[12] Healthcare Cost and Utilization Project (HCUP) Overview of the National (Nationwide) Inpatient Sample (NIS). https://www.hcup-us.ahrq.gov/nisoverview.jsp. Updated August 2018. Accessed May 10, 2018.

[13] Houchens R, Ross D, Elixhauser A Using the HCUP National Inpatient Sample to Estimate Trends. HCUP Methods Series Report #2006-05. Rockville, MD: Agency for Healthcare Research and Quality; 2015 https://www.hcup-us.ahrq.gov/reports/methods/2006_05_NISTrendsReport_1988-2004.pdf. Accessed May 10, 2018.

[14] Toyoda N, Chikwe J, Itagaki S, Gelijns AC, Adams DH, Egorova NN Trends in infective endocarditis in California and New York state, 1998-2013. JAMA. 2017;317(16):1652-1660. doi:10.1001/jama.2017.4287 28444279 PMC5470417

[15] Centers for Disease Control and Prevention. *International Classification of Diseases, Ninth Revision, Clinical Modification (ICD-9-CM)* https://www.cdc.gov/nchs/icd/icd9cm.htm. Published November 2015. Accessed May 10, 2018.

[16] Cox S, Posner SF, Kourtis AP, Jamieson DJM Hospitalizations with amphetamine abuse among pregnant women. Obstet Gynecol. 2008;111(2 Pt 1):341-347. doi:10.1097/01.AOG.000300377.82722.ad 18238971

[17] Chronic Conditions Data Warehouse. Mental health, and potentially disabling conditions - drug use disorders. https://www.ccwdata.org/web/guest/condition-categories. Published November 2017. Accessed August 24, 2018.

[18] Substance Abuse and Mental Health Services Administration.*Drug Abuse Warning Network*, *2011: National Estimates of Drug-Related Emergency Department Visits.* Rockville, MD: Substance Abuse and Mental Health Services Administration; 2013 https://www.samhsa.gov/data/sites/default/files/DAWN2k11ED/DAWN2k11ED/DAWN2k11ED.pdf. Accessed August 15, 2018.

[19] Substance Abuse and Mental Health Services Administration. Emergency department visits involving attention deficit/hyperactivity disorder stimulant medications. *The DAWN Report* January 24, 2013 https://www.samhsa.gov/data/sites/default/files/DAWN073/DAWN073/sr073-ADD-ADHD-medications.htm. Accessed May 10, 2018. 27631057

[20] Cooper HLF, Brady JE, Ciccarone D, Tempalski B, Gostnell K, Friedman SR Nationwide increase in the number of hospitalizations for illicit injection drug use-related infective endocarditis. Clin Infect Dis. 2007;45(9):1200-1203. doi:10.1086/522176 17918083 PMC2567828

[21] Heslin K, Elixhauser A, Steiner C Hospitalizations Involving Mental and Substance Use Disorders Among Adults, 2012. Rockville, MD: Agency for Healthcare Research and Quality; 2015 https://www.hcup-us.ahrq.gov/reports/statbriefs/sb191-Hospitalization-Mental-Substance-Use-Disorders-2012.pdf. Accessed May 8, 2018. 26290939

[22] Healthcare Cost and Utilization Project (HCUP) Clinical Classifications Software (CCS) for ICD-9-CM. https://www.hcup-us.ahrq.gov/toolssoftware/ccs/ccs.jsp. Published March 2017. Accessed February 28, 2018.

[23] Weiss A, Elixhauser A, Barrett M, Steiner C, Bailey M, O’Malley L Opioid-Related Inpatient Stays and Emergency Department Visits by State, 2009-2014. Rockville, MD: Agency for Healthcare Research and Quality; 2016 https://www.hcup-us.ahrq.gov/reports/statbriefs/sb219-Opioid-Hospital-Stays-ED-Visits-by-State.pdf. Accessed May 10, 2018.

[24] Compton WM, Jones CM, Baldwin GT Relationship between nonmedical prescription-opioid use and heroin use. N Engl J Med. 2016;374(2):154-163. doi:10.1056/NEJMra1508490 26760086 PMC11784537

[25] Han B, Compton WM, Blanco C, Crane E, Lee J, Jones CM Prescription opioid use, misuse, and use disorders in U.S. adults: 2015 National Survey on Drug Use and Health. Ann Intern Med. 2017;167(5):293-301. doi:10.7326/M17-0865 28761945

[26] Seth P, Scholl L, Rudd RA, Bacon S Overdose deaths involving opioids, cocaine, and psychostimulants — United States, 2015–2016. MMWR Morb Mortal Wkly Rep. 2018;67(12):349-358. doi:10.15585/mmwr.mm6712a1 29596405 PMC5877356

[27] Bureau of Labor Statistics. Table 24. Historical Consumer Price Index for All Urban Consumers (CPI-U): U.S. city average, all items. https://www.bls.gov/cpi/tables/historical-cpi-u-201710.pdf. Published October 2017. Accessed May 10, 2018.

[28] Barrett M, McCarty J, Coffey R, Levit K Population Denominator Data for Use With HCUP Databases (Updated With 2015 Population Data). Rockville, MD: US Agency for Healthcare Research and Quality; 2016.

[29] Khera R, Angraal S, Couch T, Adherence to methodological standards in research using the National Inpatient Sample. JAMA. 2017;318(20):2011-2018. doi:10.1001/jama.2017.17653 29183077 PMC5742631

[30] von Elm E, Altman DG, Egger M, Pocock SJ, Gøtzsche PC, Vandenbroucke JP; STROBE Initiative The Strengthening the Reporting of Observational Studies in Epidemiology (STROBE) statement: guidelines for reporting observational studies. PLoS Med. 2007;4(10):e296. doi:10.1371/journal.pmed.0040296 17941714 PMC2020495

[31] Härtel-Petri R, Krampe-Scheidler A, Braunwarth W-D, Evidence-based guidelines for the pharmacologic management of methamphetamine dependence, relapse prevention, chronic methamphetamine-related, and comorbid psychiatric disorders in post-acute settings. Pharmacopsychiatry. 2017;50(3):96-104. doi:10.1055/s-0043-105500 28445899

[32] Ben-Yehuda O, Siecke N Crystal methamphetamine: a drug and cardiovascular epidemic. JACC Heart Fail. 2018;6(3):219-221. doi:10.1016/j.jchf.2018.01.004 29496023

[33] Sliman S, Waalen J, Shaw D Methamphetamine-associated congestive heart failure: increasing prevalence and relationship of clinical outcomes to continued use or abstinence. Cardiovasc Toxicol. 2016;16(4):381-389. doi:10.1007/s12012-015-9350-y 26661075

[34] Ronan MV, Herzig SJ Hospitalizations related to opioid abuse/dependence and associated serious infections increased sharply, 2002–12. Health Aff (Millwood). 2016;35(5):832-837. doi:10.1377/hlthaff.2015.1424 27140989 PMC5240777

[35] Grant BF, Chou SP, Saha TD, Prevalence of 12-month alcohol use, high-risk drinking, and *DSM-IV* alcohol use disorder in the United States, 2001-2002 to 2012-2013: results from the National Epidemiologic Survey on alcohol and related conditions. JAMA Psychiatry. 2017;74(9):911-923. doi:10.1001/jamapsychiatry.2017.2161 28793133 PMC5710229

[36] Mohlman MK, Tanzman B, Finison K, Pinette M, Jones C Impact of medication-assisted treatment for opioid addiction on Medicaid expenditures and health services utilization rates in Vermont. J Subst Abuse Treat. 2016;67:9-14. doi:10.1016/j.jsat.2016.05.002 27296656

[37] Rawson RA, Gonzales R, Brethen P Treatment of methamphetamine use disorders: an update. J Subst Abuse Treat. 2002;23(2):145-150. doi:10.1016/S0740-5472(02)00256-8 12220612

[38] Roll JM, Petry NM, Stitzer ML, Contingency management for the treatment of methamphetamine use disorders. Am J Psychiatry. 2006;163(11):1993-1999. doi:10.1176/ajp.2006.163.11.1993 17074952

[39] Courtney KE, Ray LA Methamphetamine: an update on epidemiology, pharmacology, clinical phenomenology, and treatment literature. Drug Alcohol Depend. 2014;143:11-21. doi:10.1016/j.drugalcdep.2014.08.003 25176528 PMC4164186

[40] Kolodny A, Courtwright DT, Hwang CS, The prescription opioid and heroin crisis: a public health approach to an epidemic of addiction. Annu Rev Public Health. 2015;36(1):559-574. doi:10.1146/annurev-publhealth-031914-122957 25581144

[41] Patrick SW, Schiff DM; Committee on Substance Use and Prevention A public health response to opioid use in pregnancy. Pediatrics. 2017;139(3):e20164070. doi:10.1542/peds.2016-4070 28219965

[42] Rich JD, Adashi EY Ideological anachronism involving needle and syringe exchange programs: lessons from the Indiana HIV outbreak. JAMA. 2015;314(1):23-24. doi:10.1001/jama.2015.6303 26000661 PMC4496270

[43] Wakeman SE Another senseless death — the case for supervised injection facilities. N Engl J Med. 2017;376(11):1011-1013. doi:10.1056/NEJMp1613651 28296603

[44] Dasgupta N, Beletsky L, Ciccarone D Opioid crisis: no easy fix to its social and economic determinants. Am J Public Health. 2018;108(2):182-186. doi:10.2105/AJPH.2017.304187 29267060 PMC5846593

[45] Center for Behavioral Health Statistics and Quality, Substance Abuse and Mental Health Services Administration, US Department of Health and Human Services. Results from the 2016 National Survey on Drug Use and Health: detailed tables (Table 1.77A). https://www.samhsa.gov/data/sites/default/files/NSDUH-DetTabs-2016/NSDUH-DetTabs-2016.htm#tab1-77A. Accessed July 30, 2018.

[46] Center for Behavioral Health Statistics and Quality, Substance Abuse and Mental Health Services Administration, US Department of Health and Human Services. Results from the 2016 National Survey on Drug Use and Health: detailed tables (Table 1.90A). https://www.samhsa.gov/data/sites/default/files/NSDUH-DetTabs-2016/NSDUH-DetTabs-2016.htm#tab1-90A. Accessed July 30, 2018.

[47] Campo J Maternal and Newborn Inpatient Stays With a Substance Use or Use-Related Diagnosis. Olympia, WA: Health Care Research Center, Washington State Office of Financial Management; 2016 https://www.ofm.wa.gov/sites/default/files/public/legacy/researchbriefs/2016/brief075.pdf. Accessed May 10, 2018.

[48] Chalmers J, Lancaster K, Hughes C The stigmatisation of ‘ice’ and under-reporting of meth/amphetamine use in general population surveys: a case study from Australia. Int J Drug Policy. 2016;36:15-24. doi:10.1016/j.drugpo.2016.06.001 27450550

