# Supplementary material for: Evaluation of Amphetamine-Related Hospitalizations and Associated Clinical Outcomes and Costs in the United States
Source: JAMA Netw Open. 2018 Oct 19;1(6):e183758. doi: 10.1001/jamanetworkopen.2018.3758 (PMC6324446; doi:10.1001/jamanetworkopen.2018.3758)
Supplement: Supplement. — eTable 1. International Classification of Disease, 9th Edition, Clinical Modification (ICD-9-CM) Codes for Other Substance-Related Categories eTable 2. Top 15 Primary Diagnoses Among Amphetamine-Related Hospitalizations, United States - 2014/2015 [file jamanetwopen-1-e183758-s001.pdf]

Supplementary Online Content

Winkelman TNA, Admon LK, Jennings L, Shippee ND, Richardson CR, Bart G. Evaluation of amphetamine-related hospitalizations and associated clinical outcomes and costs in the United States. *JAMA Netw Open.* 2018;1(6):e183758. doi:10.1001/jamanetworkopen.2018.3758

eTable 1. International Classification of Disease, 9th Edition, Clinical Modification (ICD-9-CM) Codes for Other Substance-Related Categories

eTable 2. Top 15 Primary Diagnoses Among Amphetamine-Related Hospitalizations, United States - 2014/2015

This supplementary material has been provided by the authors to give readers additional information about their work.

©2018 Winkelman TNA et al. *JAMA Network Open.*

eTable 1. International Classification of Disease, 9th Edition, Clinical Modification (ICD-9-CM) Codes for Other Substance-Related Categories

Substance use category	ICD-9-CM diagnosis codes
Alcohol	291.0, 291.1, 291.2, 291.3, 291.4, 291.5, 291.81, 291.82, 291.89, 291.9, 303.00-303.03, 303.90-303.93, 305.00-305.03, 357.5, 425.5, 535.30, 535.31, 571.0, 571.1, 571.2, 571.3, and E860.0
Cannabis	304.3X and 305.2X
Opioid	304.0X, 304.7X, 305.5X, 965.00-965.02, 965.09, E850.0-E850.2, E935.0-E935.2, and E940.1
Cocaine	304.2X, 305.6X, and 970.81
Hallucinogens	304.5X, 305.3X, 969.6, E854.1, and E939.6
Sedatives, tranquilizers, and hypnotics	304.1X, 305.4X, 967.X. 969.4, E851, E852.X, and E853.2
Other drugs	304.60-304.63, 304.80-304.83, 304.90-304.93, 305.90-305.93, 648.30-648.34, V65.42
Drug-induced mental disorder	292.0, 292.11, 292.12, 292.2, 292.81-292.85, 292.89, and 292.9

©2018 Winkelman TNA et al. *JAMA Network Open.*

eTable 2. Top 15 Primary Diagnoses Among Amphetamine-Related Hospitalizations, United States - 2014/2015

Rank	Primary diagnosis category[a]	Weighted percent (95% CI)
1	Mood disorder	13.9 (13.1-14.8)
2	Schizophrenia and other psychotic disorder	10.2 (9.1-11.6)
3	Substance-induced mental disorder[b]	6.0 (5.5-6.5)
4	Septicemia (except in labor)	5.8 (5.5-6.2)
5	Skin and subcutaneous tissue infection	4.3 (4.1-4.5)
6	Poisoning by amphetamines[b]	4.1 (3.9-4.3)
7	Congestive heart failure (nonhypertensive)	3.4 (3.2-3.7)
8	Substance-related disorder	3.0 (2.7-3.3)
9	Alcohol-related disorder	2.7 (2.5-2.9)
10	Diabetes mellitus with complications	2.6 (2.5-2.8)
11	Poisoning by other medications and drugs	2.0 (1.9-2.2)
12	Poisoning by psychotropic agents	1.8 (1.6-1.9)
13	Amphetamine dependence or abuse[b]	1.7 (1.6-1.9)
14	Acute cerebrovascular disease	1.6 (1.5-1.7)
15	Acute and unspecified renal failure	1.5 (1.4-1.6)

[a]Primary diagnosis categories based on the Healthcare Cost and Utilization Project's Clinical Classification Software.
[b]Poisoning by amphetamine (969.72), amphetamine dependence or abuse (304.40-304.42, 305.70-305.72), and substance-induced mental disorders (292.0, 292.11, 292.12, 292.2, 292.81-292.85, 292.89, 292.9) based on International Classification of Disease, 9th edition, Clinical Modification codes.

©2018 Winkelman TNA et al. *JAMA Network Open.*
